# Genome-wide inference of regulatory networks in *Streptomyces coelicolor*

**DOI:** 10.1186/1471-2164-11-578

**Published:** 2010-10-18

**Authors:** Marlene Castro-Melchor, Salim Charaniya, George Karypis, Eriko Takano, Wei-Shou Hu

**Affiliations:** 1 Department of Chemical Engineering and Materials Science, University of Minnesota, 421 Washington Avenue SE, Minneapolis, MN 55455, USA; 2 Department of Computer Science and Engineering, University of Minnesota, 200 Union Street SE, Minneapolis, MN 55455, USA; 3 Department of Microbial Physiology, Groningen Biomolecular Sciences and Biotechnology Institute, University of Groningen, Kerklaan 30, 9751 NN Haren, The Netherlands; 4 Genentech, Inc, 1 Antibody Way, Oceanside, CA 92056, USA

## Abstract

**Background:**

The onset of antibiotics production in *Streptomyces *species is co-ordinated with differentiation events. An understanding of the genetic circuits that regulate these coupled biological phenomena is essential to discover and engineer the pharmacologically important natural products made by these species. The availability of genomic tools and access to a large warehouse of transcriptome data for the model organism, *Streptomyces coelicolor*, provides incentive to decipher the intricacies of the regulatory cascades and develop biologically meaningful hypotheses.

**Results:**

In this study, more than 500 samples of genome-wide temporal transcriptome data, comprising wild-type and more than 25 regulatory gene mutants of *Streptomyces coelicolor *probed across multiple stress and medium conditions, were investigated. Information based on transcript and functional similarity was used to update a previously-predicted whole-genome operon map and further applied to predict transcriptional networks constituting modules enriched in diverse functions such as secondary metabolism, and sigma factor. The predicted network displays a scale-free architecture with a small-world property observed in many biological networks. The networks were further investigated to identify functionally-relevant modules that exhibit functional coherence and a consensus motif in the promoter elements indicative of DNA-binding elements.

**Conclusions:**

Despite the enormous experimental as well as computational challenges, a systems approach for integrating diverse genome-scale datasets to elucidate complex regulatory networks is beginning to emerge. We present an integrated analysis of transcriptome data and genomic features to refine a whole-genome operon map and to construct regulatory networks at the cistron level in *Streptomyces coelicolor*. The functionally-relevant modules identified in this study pose as potential targets for further studies and verification.

## Background

Streptomycetes are soil-living organisms with a complex life cycle that includes formation of aerial mycelia and spores. Members of this genus have large genomes and the capability of producing multiple secondary metabolites, many of which have uses as antibiotics, anti-tumor agents, and immunosuppressants [1]. The genome of *Streptomyces coelicolor*, the model organism for this high G+C genus, contains 7825 genes. The genome contains more than 20 secondary metabolite clusters and 965 genes encoding proteins predicted to have a regulatory role [2].

With more genes than lower eukaryotes and an unusually high number of regulators, deciphering the regulatory network of *Streptomyces coelicolor *remains a challenge. Regulation is a dynamic process, in which overlapping signaling cascades integrate into complex networks, linking diverse aspects of growth, morphology, and secondary metabolite production. In addition, in the case of bacteria, genes can be co-transcribed as polycistrons, and it is at this level of cistrons that regulation occurs, rather than at the level of individual genes.

Single knock-out/disruption mutations have been extensively used in this organism to try to decipher the mechanisms regulating secondary metabolite production and their link to morphological changes. The study of these mutants has made multiple advances over the years, including the characterization of the regulators of gene clusters specific to synthesis of antibiotics. These approaches have also revealed that cross-regulation among disparate pathways occur [3], and is thus desirable to explore regulation at a genome scale. Transcriptome profiles across a diverse set of conditions can be used to systematically determine regulatory interactions [4].

In this study we used functional similarity, conservation of gene order, intergenic distance, and gene expression similarity as features for refining our previously published operon predictions [5]. Gene expression data at the cistron level was then used to predict networks centered on 692 regulatory cistrons.

Among the algorithms to reconstruct whole genome regulatory networks, the information-theoretic approaches have gained support in the bioinformatics community. These approaches rely on the estimation of mutual information (MI) from expression data between pairs of genes, or cistrons, to estimate candidate interactions. MI is a correlation measure that can detect non-linear correlations that other measurements like Euclidean distance or Pearson correlation cannot identify. Among the state-of-the-art information-theoretic approaches are relevance networks [6], ARACNE [7,8], CLR [4], and MRNET [9]. Benchmark studies [10,11] comparing the accuracy of the methods have not resulted in a clear winner over all, as the performance of the algorithms is affected by the type of network, and the mutual information estimator, among others. In this work we inferred whole-genome regulatory networks with ARACNE [7]. ARACNE removes indirect interactions by using the data process inequality (DPI), a property of MI [8,12]. ARACNE has been used to identify putative transcriptional targets of the cancer related genes MYC and KLF6 [13], and to reconstruct breast, colorectal, and glial normal and cancerous tissue gene coexpression networks [14].

## Methods

### Microarray data compilation and processing

The transcriptome data used in this analysis was obtained from in-house generated data and the public repository databases Stanford Microarray Database, Gene Expression Omnibus (GEO), and Array Express. In addition to data used previously [5] for operon prediction, 326 transcriptome data were used. The additional data consists of 105 hybridizations performed on Affymetrix diS_div712a GeneChips [15]; 55 cDNA:cDNA hybridizations [16-20]; and 166 cDNA:gDNA hybridizations [21-24] and GEO [25] accession numbers GSE21807, GSE21808, GSE21811, GSE22398, and GSE22399. The data was divided into three datasets, according to the platform used: dataset 1 for cDNA:gDNA, dataset 2 for cDNA:cDNA, and dataset 3 for Affymetrix chips. Eight transcriptome data (cDNA: cDNA) were removed before further analysis as more than 30% of the genes in those samples were flagged absent.

Prior to analysis using ARACNE, data for genes with low expression dynamics or with a large number of absent flags was removed. It was desirable that the expression data of a gene exhibit good expression dynamics in at least one of the datasets. Thus, a criterion was established that a gene must be flagged absent in less than 20% of the samples and its expression data must have a standard deviation greater than the 25% percentile of the population. This criterion must be met in at least one of the two datasets (dataset 1 or dataset 2). In addition, the expression data of the gene must satisfy a minimum passing criterion in the other dataset - flagged absent in less than 50% of the samples. Also, a probeset corresponding to that gene must be present on the Affymetrix diS_div712a GeneChip to ensure that Affymetrix gene expression data for that gene was available. The following Boolean logical criterion was used for gene selection:

(1) {(a≤0.20)AND(b≥0.50)AND(c≤0.50)AND(e=TRUE)} OR {(c≤0.20)AND(d≥0.43)AND(a≤0.50)AND(e=TRUE)}

where,

*a = *Fraction of absent flags in dataset 1

*b *= Standard deviation in dataset 1 (25^th ^percentile of the standard deviations is 0.50)

*c = *Fraction of absent flags in dataset 2

*d *= Standard deviation in dataset 2 (25^th ^percentile of the standard deviations is 0.43)

*e *= Presence of a probeset for that gene on Affymetrix diS_div712a GeneChip

In all, transcript profiles of 6225 genes, corresponding to 4399 cistrons, were used. The *k*-nearest neighbor method [26] was used to estimate any missing values, as ARACNE requires a complete expression matrix. For each of the three datasets the expression data for each gene was *z*-standardized to an average of 0 and a standard deviation of 1.

### Features used in operon prediction

Functional similarity was estimated based on the protein classification scheme available at the Welcome Trust Sanger Institute [27] and on Gene Ontology (GO) terms. In the case of the protein classification scheme, functional similarity was determined for adjacent genes if both genes were assigned to one of the 140 protein classes. A score of 1 was assigned when both genes belonged to the same functional class and -1 when they belonged to different classes.

Functional similarity based on GO terms was based on biological process and molecular function, two of the three organizing principles of Gene Ontology, and assessed with two metrics: the Czekanowski-Dice score [28] and the information theoretic metric available in the R package GoSim [29]. The Czekanowski-Dice score was calculated using the formula:

(2) $$\text{Czekanowski}-\text{Dice score=}\frac{2c}{a+b},$$

where *a *and *b *are the number of GO terms associated with each gene, and *c *is the number of GO terms common to both genes.

Conservation of gene order was estimated by the number of bacterial genomes in which the orthologs of a pair of adjacent genes are present in the same order. The number of orthologs was obtained from OperonDB [30,31] and it is included in additional file 1. Intergenic distance was calculated from data downloaded from StrepDB [32]. Pearson correlation (*r*), calculated between pairs of adjacent genes, was used as gene expression similarity measure.

### Supervised classification for operon prediction

Supervised classification models for the prediction of operons were obtained using SVM^*light *^[33]. Classifiers were assessed by a 10-fold cross-validation scheme. Recall, false positive rate and area under receiver operating characteristic (ROC) curves were used to assess the performance of classifiers as previously described [5].

Positive and negative classes were defined as known operon pairs (KOP) and non-operon pairs (NOP), respectively, as described previously [5]. The positive training set consisted of 425 KOPs. Of these KOPs, 149 were used in our previous study [5]. An additional 266 gene pairs were experimentally verified to be co-transcribed in the same study. Also, eleven pairs were identified from six recently reported operons: *nikABCDE *[34], *devAB *[35], *nrdABS *and *nrdRJ *[36], *znuACB *[37], and *rpmG3*-*rpmJ2 *[38]. This last pair, *rpmG3*-*rpmJ2 *had also been verified in our previous study [5]. The negative training set consisted of 131 NOPs. Of these NOPs, 119 gene pairs were retained from our previous study comprising 122 NOPs. The three pairs that were removed were verified to be co-transcribed in the previous report. Twelve additional NOPs were obtained from the six recently reported operons mentioned above. The list of positive and negative training sets is given in additional file 2.

### Transcriptional network prediction using ARACNE

Transcriptional networks were predicted on the whole genome using ARACNE [7,8]. The input to ARACNE consisted of a matrix containing the gene expression data at the cistron level and a list of regulators. The gene expression matrix consisted of 4399 rows, corresponding to cistrons, and 524 columns, corresponding to microarrays. A *p*-value of 1.0 × 10^-9 ^was used as threshold for mutual information. A DPI tolerance of 0.05 was used as criteria to remove possible indirect interactions. Predicted networks were visualized in Cytoscape [39] within ARACNE.

### Network modules with functional enrichment and consensus sequences

Fisher's exact test was used to identify network modules in which a significant fraction of genes are involved in the same biological pathway or function, as defined by the protein classification scheme [27] and GO terms. Those network modules with a *p*-value less than 1.0 × 10^-4 ^were considered significantly enriched. The R package *qvalue *[40] was used to calculate the corresponding *q*-values using the bootstrap option [41]. All network modules reported as significantly enriched were significant at an FDR = 0.01.

The upstream regions (300 bp) of the cistrons belonging to the same network module were examined for the presence of consensus sequences using MEME version 3.5.7 [42,43]. The zero order background Markov model used in MEME (A: 0.153; C: 0.351; G: 0.347; and T: 0.149) was determined by calculating the fraction of each base in the upstream region of all 5346 predicted cistrons. To reduce the probability that the reported motifs are not statistically significant, motifs were determined for the same sequences but after randomly shuffling the sequence letters. To make this criterion stricter, this was repeated five times. An *E*-value_threshold _was set for each network module as the minimum of five *E*-values determined when the upstream cistron sequences were randomly shuffled. A consensus sequence was considered present in a network if the *E*-value was less than the *E*-value_threshold_. Consensus sequence images were generated with WebLogo [44].

## Results

### Operon prediction refinement

Building upon the whole genome operon map developed previously [5] we employed additional features for operon prediction: functional similarity of adjacent genes, and conservation of gene order. The training set used in this work consisted of literature reported operons, and 266 experimentally verified pairs predicted from our previous work. The positive training set thus consisted of 425 known operon pairs (KOPs), while the negative training set consisted of 131 non-operon pairs (NOPs). The compiled transcriptome dataset comprised a total of 524 cell samples, substantially larger than the 206 samples used in the previous predictions.

#### Features for operon prediction

Genes which are part of an operon are often involved in the same biological function or pathway. Functional similarity was assessed for the positive and negative training sets based on a protein classification scheme available at the Welcome Trust Sanger Institute [27] and on Gene Ontology (GO) terms. Functional similarity assessment requires that both genes in a pair have a category assigned, thus not all KOPs and NOPs could be tested for functional similarity. Functional similarity based on the protein classification scheme revealed that a high percentage of KOPs (72%) corresponded to pairs in which both genes belonged to the same protein class, whereas for NOPs the percentage was low (10%). Functional similarity based on GO was calculated using the Czekanowski-Dice score (see Methods) and an information theoretic metric available in the R package GoSim [29]. A Czekanowski-Dice score greater than 0.6 was calculated for 33% of the KOPs, but for none of the NOPs. Based on the information theoretic metric, adjacent genes in 79% of the KOPs have functional similarity greater than 0.6, while only 37% of the NOPs have a similarity greater than 0.6. All these functional similarity metrics indicate that adjacent genes in the same operon have a high-likelihood of being involved in the same biological function. Therefore, these similarity metrics can be used for operon prediction.

Genes in the same operon are often conserved across multiple genomes. Conservation of gene order has been previously used for operon prediction in prokaryotes [31]. The number of bacterial genomes in which the orthologs of adjacent *Streptomyces coelicolor *genes are present in the same order was thus used as a feature for operon prediction. Also, KOPs have shorter intergenic distance compared to NOPs, and therefore, this feature was also used for operon predictions.

Genes which are part of an operon and are co-transcribed have similar expression profiles. Pearson correlation (*r*) was used as measure of gene expression similarity between the transcript profiles of pairs of adjacent genes. A correlation *r *> 0.7 was observed for 35% of the adjacent gene pairs in the KOPs. In contrast only 2% of the adjacent gene pairs in the NOPs had a correlation *r *> 0.7. The sharp discrimination between the two classes strongly indicates the importance of transcriptome data for predicting operons.

#### Classifiers to differentiate KOPs and NOPs

**Figure 1:**
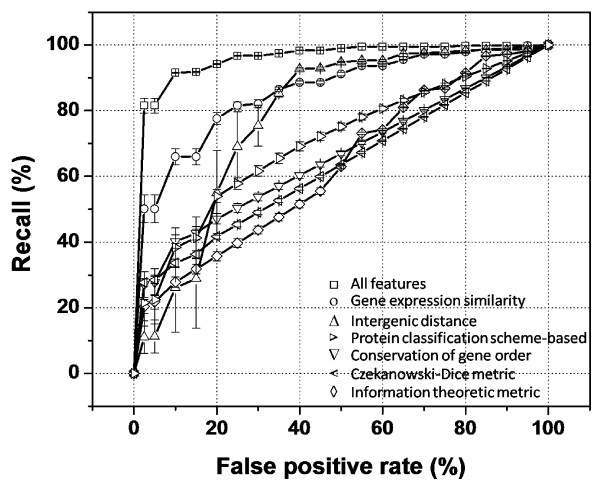
**Comparison of different SVM classifiers by 10-fold cross-validation and ROC graphs**. False positive rate is the percentage of NOPs misclassified as operon pairs. Recall is the percentage of KOPs correctly classified as operon pairs. Error bars indicate ± 1 standard deviation (*n *= 10).

Binary support vector machine (SVM) classifiers were constructed for differentiating KOPs and NOPs using the individual features described in the previous section. A classifier combining all the features was also constructed. The performance of the constructed classifiers was compared by using a 10-fold cross-validation scheme and receiver operating characteristic (ROC) curves (Figure 1). Table 1 shows a comparison of the area under ROC curves (AUC) for all the classifiers. The null hypothesis was tested by comparing the AUC of ten ROC graphs for each classifier by one-tailed paired *t*-test. The best single feature classifier is that based on gene expression similarity with an AUC of 0.87, which is better than the intergenic distance-based classifier (*p-*value = 2.8 × 10^-2^, paired *t-*test). The radial SVM model based on all the features, which has an AUC of 0.97, outperforms all the classifiers based on single features, including the gene expression similarity classifier (*p-*value = 1.6 × 10^-4^, paired *t-*test).

**Table 1:** Comparison of the AUC of different classifiers.

**No**.	Feature(s)	Average AUC	*p*-value	Null hypothesis
I	Functional similarity			
	*a. Protein classification scheme-based*	0.72		
	*b. Czekanowski-Dice score*	0.65		
	*c. Information theoretic metric*	0.65		
II	Conservation of gene order	0.68		
III	Intergenic distance	0.80		
IV	Gene expression similarity	0.87	2.8 × 10^-2^	AUC_IV _- AUC_III _= 0
V	All features	0.97	1.6 × 10^-4^	AUC_V _- AUC_IV _= 0

#### Whole genome identification of transcription units

The operon status of same-strand pairs in the genome was predicted using the SVM classifier based on all the features. The SVM model assigns a score to each same-strand gene pair. A positive score indicates that the adjacent genes are predicted to be co-transcribed. Adjacent gene pairs with positive score were grouped into operons. A total of 5346 transcription units were predicted (additional file 3). Among these, 1389 transcription units are polycistronic, containing two or more genes.

### Whole genome regulatory network prediction using ARACNE

Gene expression regulation occurs in prokaryotes at the level of cistrons instead of individual genes. The predicted cistrons were used as the basis to infer regulatory networks using ARACNE (Algorithm for the Reconstruction of Accurate Cellular Networks) [7,8]. The interactions predicted with ARACNE were of the type "cistron A regulates target cistron B". Cistrons containing at least one gene encoding a regulatory protein were categorized as "cistron A". The regulatory proteins belong to families such as sigma factors, transcription factors, DNA-binding proteins, two-component systems, defined-family regulators, and repressors. ARACNE was used to compare the expression of every combination of two cistrons to identify the pairs with statistically significant and high mutual information. ARACNE infers regulatory interactions when pairs exhibit a high degree of expression dependency or correlation. Indirect interactions are eliminated by using the data processing inequality (DPI).

ARACNE predicts interactions based on a matrix of expression values and a list of regulators. To generate the matrix of expression values the profiles of all 7825 genes from the 524 transcriptome samples were examined and those with low dynamic expression profiles were removed. In all, the expression profiles of 6225 genes, corresponding to 4399 cistrons, were used for network prediction. The expression values for cistrons were obtained by averaging the expression values of adjacent genes in the same predicted cistron over 524 transcriptome samples. Of the 4399 cistrons, 692 contain at least one gene encoding a putative regulator. These 692 cistrons constituted the input list of regulators to ARACNE.

**Figure 2:**
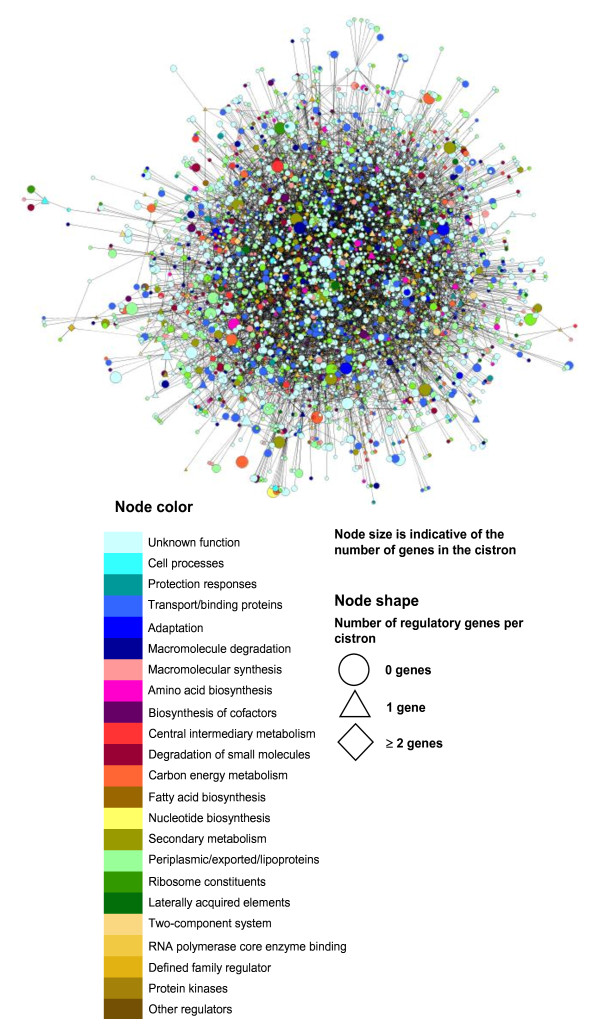
**Predicted transcriptional regulatory network for *S. coelicolor***. Each node corresponds to a cistron and every edge represents a regulatory interaction between two nodes. The entire network comprises 3527 nodes and 7170 edges.

Using a *p*-value of 1.0 × 10^-9 ^as the threshold for mutual information (MI) and a data processing inequality (DPI) tolerance of 0.05, a total of 7170 interactions between 3527 cistrons were identified by ARACNE. For each of the 692 cistrons encoding one or more regulatory proteins, a "network module" was predicted. A network module is comprised of cistrons predicted to be transcriptionally controlled by the regulatory protein, also referred to as the "hub" of the module. The complete predicted network containing 692 interconnected network modules is shown in Figure 2. Each node represents one cistron and edges between two nodes represent a potential interaction. The detailed resulting matrix for the complete network is given on additional file 4, in which the MI scores between interacting cistrons are given. The global connectivity properties of the network can be described by a power-law relationship given by *p *= 46.6 × *k *^**-2.71 **^where, *p *is the probability that a regulatory node has *k *interactions. This is indicative of a scale-free network structure. A small fraction of the regulatory nodes are highly connected and they account for a large number of interactions. Each of the top four hubs (SCO0588, SCO3063, SCO3986, and SCO5454-SCO5455) interacts with more than 50 cistrons. Interestingly, three (SCO0588, SCO3986, and SCO5454-SCO5455) of these four hubs encode two-component systems that regulate gene expression by sensing environmental cues.

This result is highly encouraging, as the mode of action of two-component systems involves phosphorylation and not only interactions at transcript level. Nevertheless some interactions can be inferred from transcript levels. For two-component systems such interaction could be the effect of autoregulation, as has been reported in *Streptomyces *(AbsA [45]) and other organisms (TrcRS [46], SenX3-RegX3 [47], PrrAB [48]).

### Supporting evidence for predicted network modules

#### Network modules containing known edges

**Figure 3:**
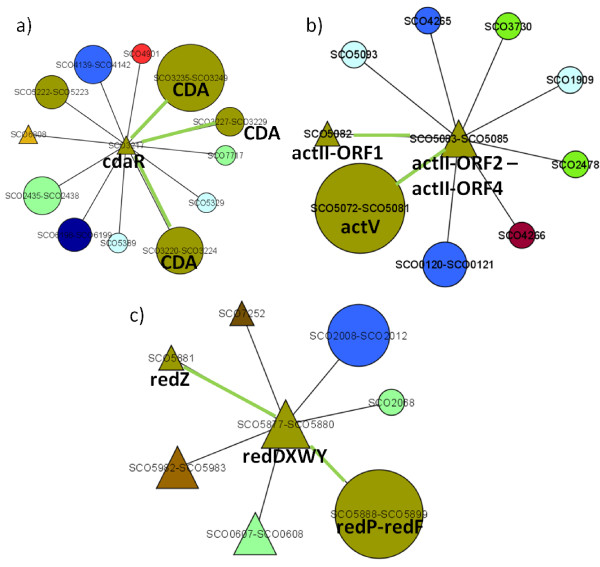
**Some network modules with known edges**. a) *cdaR *and the CDA cluster; b) *actII-ORF4 *and the ACT cluster; c) *redD *and *redZ*, and *redZ *and the RED cluster. Green edge lines indicate known interactions. Node shapes and colors are as indicated in Figure 2.

**Figure 4:**
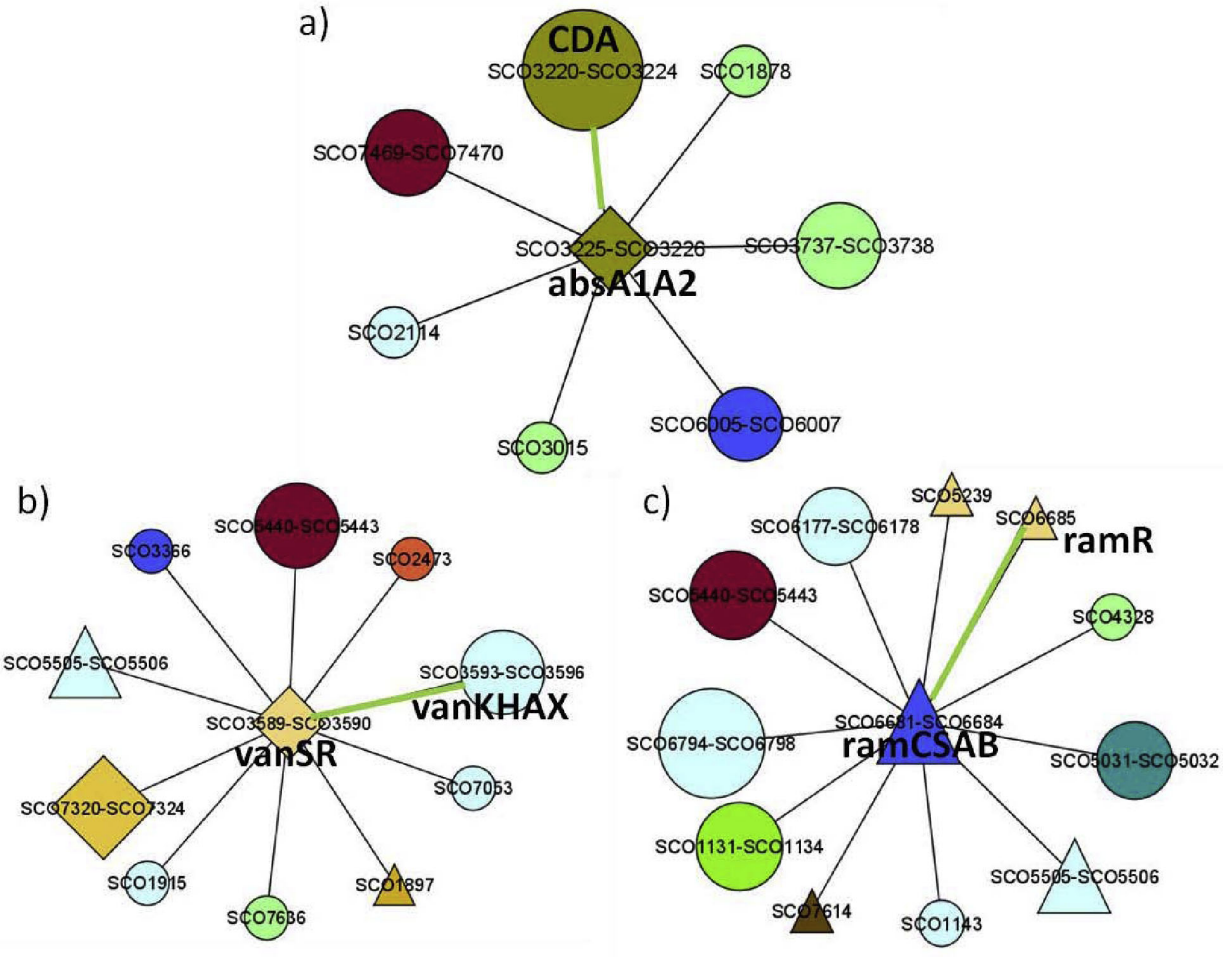
**Additional network modules with known edges**. a) The two-component system (TCS) operon *absA1A2 *and the CDA cluster; b) The TCS operon *vanRS *and the vancomycin resistance operon *vanKHAX*; c) *ramR *and *ramCSAB*. Green edge lines indicate known interactions. Node shapes and colors are as indicated in Figure 2.

The predicted interactions include known interactions that have been reported in previous studies, giving credence to this prediction. Among the known interactions retrieved are those between *cdaR *[49,50], *actII-ORF4 *[51] and their corresponding putative clusters (CDA and actinorhodin, respectively). The multilevel regulatory mechanism involving RedZ activating *redD *[52], in turn activating the RED biosynthetic cluster was also retrieved in our results. The network modules containing these interactions are shown in Figure 3. Known interactions involving two-component systems were also predicted, for example the two-component system AbsA1-AbsA2 acting on the CDA cluster [53], and VanRS acting on *vanKHAX *[54]. Another known interaction retrieved in our predictions is that between *ramR *and *ramCSAB *[55], which are required for production of aerial hyphae. The network modules containing these interactions are shown in Figure 4.

#### Identification of consensus sequences

Operons which are part of the same regulon (i.e., operons activated or repressed by a common regulatory protein), often have a consensus sequence in their upstream region. Consensus sequences have been used not only for regulon prediction, but for operon prediction [56]. For each network module, the upstream regions (300 bp) of the cistrons in that module were examined for the presence of consensus sequences using MEME [42,43]. Consensus sequences in 414 network modules were identified. In 84 of those network modules, the consensus sequence appeared in the upstream region of all the network module elements. Additional file 5 lists the consensus sequences found in each network module.

#### Network modules containing known consensus sequences

Several previous reports on *Streptomyces coelicolor *have identified the upstream consensus binding site of regulatory proteins. The consensus sequences discovered in this study were compared with previously reported binding sites. Overlaps between discovered consensus sequences and previously reported binding sites strengthen the evidence for the validity of our predicted network modules. Some of the commonalities between the sequences discovered in this study and those previously reported, are presented next. We also report the presence of these consensus sequences in additional network module members.

**Figure 5:**
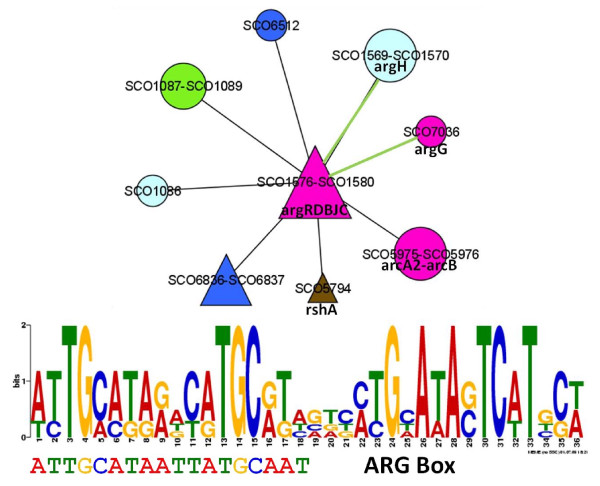
**Arginine related network module**. Network module 151 centered on *argRDBJC*. The discovered consensus sequence (top) is shown aligned to the previously described ARG box (bottom). Node shapes and colors are as indicated in Figure 2.

An ARG box has been reported in the upstream region of the *Streptomyces clavuligerus *genes *argG *and *argC *[57]. A consensus sequence was discovered in the upstream region of some elements of network module 151, centered on *argRDBJC*. The consensus sequence was identified not only on *argG *and *argC*, but also on *argH *and SCO1086. The previously reported ARG box is shown aligned with the consensus sequence found for network module 151(also shown) in Figure 5.

**Figure 6:**
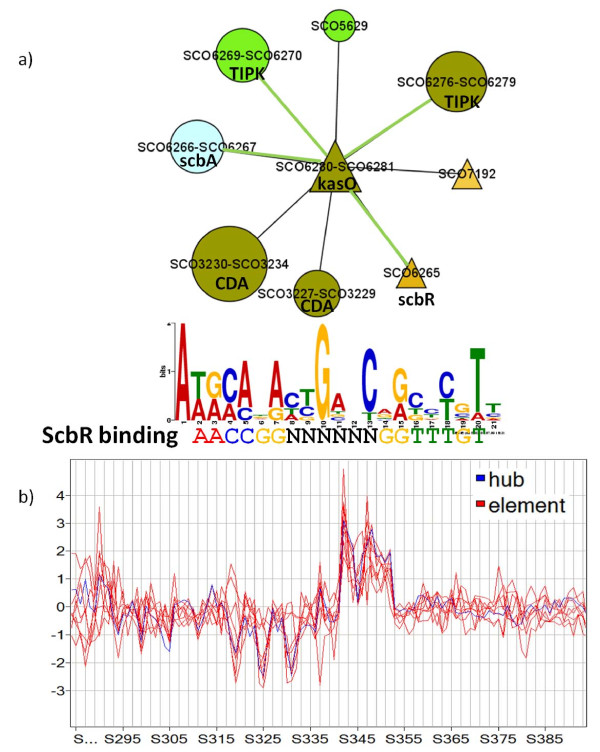
**TIPK related network module**. a) Network module 544 centered on *kasO*. The discovered consensus sequence (top) is shown aligned to the previously described ScbR binding motif (bottom). b) Partial expression profile along 100 microarrays for the 9 cistrons belonging to network 544 (Number of microarrays limited for plotting purposes). Green edge lines indicate known interactions. Node shapes and colors are as indicated in Figure 2.

An ScbR binding motif has been reported in the intergenic region of *scbA*-*scbR*, and the upstream region of *kasO *[58]. The reconstructed network module 544 centered on *kasO *contains eight interactions, including interactions with *scbA *and *scbR*. A consensus sequence was found in the upstream region of all nine elements of this network module. The previously reported ScbR binding consensus sequence is shown aligned with the consensus sequence found for network module 544 (also shown) in Figure 6. Also in the figure are partial gene expression profiles for the network module members along 100 microarray samples (number of microarrays limited for plotting purposes only). The strong correlation between the regulatory hub (*kasO*) and the eight module elements is evident.

**Figure 7:**
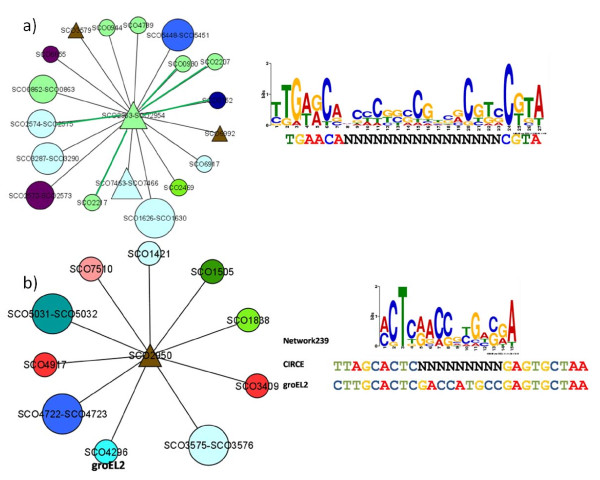
**Additional network modules with known consensus sequences**. a) Network module 240 centered on *sigU*. The discovered consensus sequence (top) is shown aligned to the previously described *sigU *binding motif (bottom); b) Network module 239. The discovered consensus sequence (top) is shown aligned to CIRCE (middle) and the upstream region of *groeL2 *(bottom). Green edge lines indicate known interactions. Node shapes and colors are as indicated in Figure 2.

Additional commonalities between consensus sequences in other network modules and previously reported motifs were found. For example, the *sigU*-dependent promoter sequence TGA[AG]C[AG][N_16-17_]CGTA [59] is similar to the consensus sequence identified in the *sigU*-centered network module 240. An overlap was also detected for CIRCE (Controlling Inverted Repeat of Chaperone Expression) [60], known to be present in the upstream region of *groEL2*, and the consensus sequence discovered in all the upstream regions of the *groEL2 *containing network module 239. These network modules and the discovered consensus sequence aligned to previously reported motifs are shown in Figure 7.

#### Identification of biologically enriched network modules

A functional module is a group of components and their interactions that can be attributed a specific biological function [61]. We investigated which network modules represented functionally coherent modules. Fisher's exact test was used to identify the network modules in which a significantly larger number of members were associated with a protein class or a GO term than would be associated by chance. The protein classification used was that at the Welcome Trust Sanger Institute [27]. At a *p*-value threshold of 1.0 × 10^-4^, 146 network modules were enriched in 33 different protein classes. Twenty-five network modules were enriched in the secondary metabolism protein class. Additionally, 16 network modules were enriched in the polyketide synthase protein class. For the classification using GO terms, at a *p*-value threshold of 1.0 × 10^-4^, 115 network modules were enriched in 67 GO terms. The term that appeared as enriched in the most number of network modules (13) was NADH dehydrogenase (ubiquinone) activity. The complete list of 188 unique enriched network modules can be found in additional file 6.

### Functionally coherent network modules including a consensus sequence

Functionally coherent network modules that also contain a consensus sequence are highly probable to indicate true interactions. Thus, in the above functionally coherent modules, those containing a consensus sequence were identified. A total of 20 network modules contain a consensus sequence in the upstream region of all of its members and present biological enrichment (additional file 7). Of those network modules, 8 were identified as enriched in both a protein class and a GO term (Table 2).

**Table 2:** Network modules enriched and with consensus sequences

Network	Regulator	Protein class enriched	GO terms enriched
20	SCO0233	Secondary metabolism	ATPase activity, coupled to transmembrane movement of substances

45	SCO0453	Transport/binding proteins	Hydrolase activity, hydrolyzing O-glycosyl compounds
			Transporter activity
			Transport

49	SCO0487	Sigma factor	DNA binding
			Transcription initiation
			Sigma factor activity

56	SCO0588	Anaerobic respiration	Electron transporter activity
		Electron transport	Mitochondrial electron transport, NADH to ubiquinone
		Fatty acid and phosphatidic acid biosynthesis	NADH dehydrogenase (ubiquinone) activity
			Nitrate reductase activity
			Nitrate reductase complex

431	SCO4920	Sigma factor	Transcription initiation
			Sigma factor activity

544	SCO6280	Secondary metabolism	Ligase activity
			Cofactor binding

636	SCO7325	Adaptations, atypical conditions	Structural molecule activity

691	SCO7808	Cobalamin	Methyltransferase activity
			Biosynthetic process

**Figure 8:**
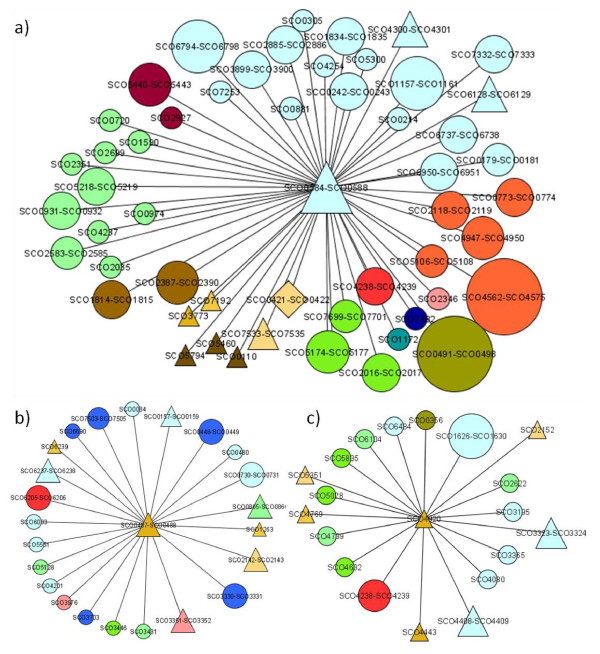
**Network modules enriched and with a consensus sequence in all their members**. Network modules are enriched in both a protein class and a GO term. a) Network module 56 centered on SCO0588, b) Network module 49 centered on SCO0487, c) Network module 431 centered on SCO4920. Node shapes and colors are as indicated in Figure 2.

Among the network modules with a consensus sequence in all of their members and biological enrichment in both a protein class and a GO term are network modules 56, 49, and 431. Network module 56 (Figure 8a) is the network module with the highest number of connections (53). Network module 49 (Figure 8b) is centered on a putative MarR family transcriptional regulator. A third of the 24 cistrons of network module 49 contain regulatory genes, including some that can coordinate cellular responses to external signals (e. g., MarR regulator, two-component system, and extracytoplasmic function sigma factors). It is possible that network module 49 is involved in responding to an environmental change that is currently unknown. Network module 431 (Figure 8c) is centered on a putative deoR family transcriptional regulator. More than one third of the 20 cistrons in network module 431 include regulatory genes, like the morphology-related *bldN*. This network includes conservon *cvnABCDE9*, a probable membrane-associated complex which may connect to the *bld *cascade [62]. Network module 431 could thus be involved in detecting nutritional limitation and the transition from substrate to aerial mycelia. Other network modules that present a consensus sequence in all their members and enrichment in a protein class and a Gene Ontology term appear in additional file 8.

## Discussion

In this study, we integrate large scale transcriptome data with genomic features to predict operons in the antibiotic producer *Streptomyces coelicolor*. The transcriptome data, at the cistron level, was then used to infer the whole genome regulatory network of this organism. The network modules, centered on cistrons containing genes encoding regulatory proteins, contain potential interactions between genes encoding regulatory proteins and their targets. Some of the interactions in the network modules correspond to experimentally known interactions. In addition, the network modules were analyzed for functional enrichment and the presence of consensus sequences. Some of the consensus sequences overlap previously described binding sequences and motifs.

### Improved operon prediction by using an expanded transcriptome set

The inclusion of additional predictive features and the expansion of the training set and the transcriptome dataset resulted in an improvement in the operon predictability of the classifiers developed in this work. The performance of the classifier based on gene expression similarity, as determined by area under ROC graph, improved from 0.81 in the previous work to 0.87 in this study. Even though the classifiers based on functional similarity performed poorly, most likely due to the lack of GO term assignment for many genes, they contributed to improve the performance of the classifier including all features. The area under ROC graph increased from 0.91 in the previous work to 0.97 in this study. Comparison of our current operon predictions to our previous predictions indicated a good agreement between the two sets. Of the 4965 same strand pairs, 4439 (89.4%) retained the same prediction. Of the 526 differences, 422 correspond to adjacent genes predicted to be co-transcribed in the current prediction, but not in the previous one. Only 104 differences corresponded to adjacent genes not predicted as co-transcribed in the current work and predicted as co-transcribed in the previous work. Most of these pairs had a low expression correlation in the expanded dataset.

The training set for the current predictions consisted of 425 KOPs and 131 NOPs. The KOPs consist of literature-reported operons as well as those experimentally verified in our previous study [5]. Thus, the training set contains more than three-fold higher KOPs compared to NOPs creating the possibility of an imbalance between the positive and the negative training sets in the operon model. However, as noted above, with an 89.4% overlap, there is a high degree of consistency between the prediction of the previously-reported model and the current predictions. The previous predictions employed a more balanced training set (149 KOPs and 122 NOPs) and the prediction results were experimentally verified. Thus, the consistency between the two predictive models gives credence to the results of the current predictions.

### Reverse engineering transcriptional network prediction

An advantage of the algorithm employed in this study (ARACNE) is that unlike clustering algorithms (such as *k-*means, or self-organizing maps) where cistrons or genes are assigned to mutually exclusive groups, a cistron can participate in multiple network modules, thus linking them and allowing a cistron to engage in different biological functions. ARACNE identified a *Streptomyces coelicolor *transcriptional network with scale-free connectivity distribution. Scale-free architecture has been noted in other networks derived from transcriptional interactions [7], metabolic reactions [63], and protein-protein interactions [64].

### Additional considerations for regulatory predictions

An implicit assumption of most reverse engineering approaches based on microarray data is that all the network components are fully observed. However gene interactions are not static and additional layers of gene regulation exist. Although on a global level, mRNA abundance correlates with the protein levels of the corresponding genes, discrepancies between mRNA and protein profiles have been noted for several genes in *Streptomyces coelicolor *[65]. Moreover, due to post-translational modifications (e.g., phosphorylation of two-component systems), the active protein levels cannot be reliably estimated from transcript levels. These uncertainties introduce hidden variables which are not observed in transcriptomic studies. Due to this limitation of partial observability, it may be impossible to identify all the direct interactions and eliminate those interactions that arise due to indirect statistical dependencies [66]. It is conceivable that many of these regulatory predictions can be substantiated and improved by combining gene expression data with other genomic data sources such as functional annotation, associations discovered by text-mining biomedical literature, and protein-protein interactions. In addition, approaches that detect dependencies between genes at different time delays are starting to emerge, see for example [67].

### Combination of network modules with annotation and consensus sequence presence

A vast majority of the 7170 interactions predicted in this study are novel and not yet experimentally verified. Because the network prediction was based only on transcriptome data at the cistron level, other interaction types involving proteins and even protein modifications would most likely not be captured with this methodology. It is encouraging though that known protein-DNA interactions were obtained in several network modules. Techniques such as EMSA, ChIP-chip and even ChIP-Seq can be used to experimentally verify these predictions. However, genome-scale experimental data for protein-DNA interactions in *Streptomyces coelicolor *is at the moment almost non existing. Prioritization of these predicted inferences will undoubtedly assist any future attempts to further analyze or verify these interactions. In this study a total of twenty network modules (additional file 7) presented functional enrichment and the presence of a consensus sequence in all of its members. These modules represent promising candidates for further analysis and experimental verification.

### Network module overlap with coherent clusters from an independent study

In a recent study by Nieselt et al. [68] the metabolic switch of *Streptomyces coelicolor *was studied by clustering of temporal transcriptome profiles, which resulted in several biologically coherent clusters, dominated by a few large operons. The eight clusters discussed in that study were compared to our predicted network modules and a considerable overlap was identified in all of them. This overlap includes the clusters associated with synthesis and regulation of the cryptic type I polyketide, and the RED and ACT antibiotics, which have several genes in common with the biologically-relevant networks identified in this study. Additionally, the ribosomal gene cluster from Nieselt et al. includes 46 genes, 23 of which appear in our network module 570, which is enriched in the protein class "Ribosomal proteins - synthesis, modification" and the GO cellular component "Ribosome". Similarly, the nitrogen metabolism cluster from Nieselt et al. includes five genes, three of which appear in our network modules 213 and 488. The network module 213 is enriched in the GO terms "nitrate reductase activity" and "nitrate reductase complex" whereas the network module 488 is enriched in the GO term "nitrogen compound metabolic process", which indicates that both networks may be involved in nitrogen metabolism. Some of the genes up regulated by phosphate depletion also appear in our network modules 370 and 371, which are centered on *phoU *and *phoRP*, respectively. Thus, the overlaps between the predicted network modules and the coherent gene clusters from an independent study further indicates the importance of combining global and temporal gene expression datasets with physiological information such as gene functions and consensus sequences.

## Conclusions

Here, we implement a systematic approach for mining large volumes of transcriptome data to predict the transcription regulatory network of *Streptomyces coelicolor*. The network comprises more than 7000 direct associations between putative transcription factors and more than 3500 predicted cistrons in *Streptomyces coelicolor*. The network displays a scale-free architecture with a small-world property observed in several biological networks in bacteria as well as higher organisms. A substantial percentage of these interactions comprise network modules with coherency of biological function. Further attempts to integrate diverse genomic dataset will seek to improve the sensitivity and specificity of these network predictions. Such integrative efforts substantiated with experimental validation present a highly promising systems approach for elucidating the regulatory determinants of secondary metabolism.

## Authors' contributions

MCM performed network interpretations, consensus sequence analysis, and manuscript writing. SC performed operon and network prediction, and assisted in manuscript writing. GK reviewed results and participated in critical discussions. ET participated in discussions. WSH supervised the study, reviewed results and assisted in manuscript preparation. All authors have read and approved the final manuscript.

## Supplementary Material

Additional file 1 **Conserved pairs.** Column 1 and 2 contain the pair of genes, column 3 the probability, and column 4 the number of genomes in which the pair is conserved. Click here for file

Additional file 2 **Training set**. Column 1 and 2 contain the pair of genes, and column 3 its status as KOP or NOP. Click here for file

Additional file 3 **Operon predictions.**  Column 1 contains genes, and column 2 contains the operon into which the gene was predicted. Click here for file

Additional file 4 **Network predictions. **Rows represent network elements, columns indicate network modules and the cistron containing the regulatory gene. Numbers are MI values, higher MI values indicate higher correlation. Click here for file

Additional file 5 **Summary of the consensus sequences found in the networks**. For each network the values *E*-value_threshold _and *E*-value are given. The number of motifs detected and if applicable its consensus sequence are given, together with the total number of elements in the module and the number of elements in which the consensus sequence was detected. Click here for file

Additional file 6 **Enriched networks.** Worksheet 1 contains the list of network modules enriched according to the protein classification scheme, and worksheet 2 those enriched according to GO terms. For each of the enriched network modules the *p*-value, *q*-value and the enriched class or term is given. All network modules are significant at an FDR = 0.01. Click here for file

Additional file 7 **Details of the 20 network modules enriched in both a protein class and a GO term**. The number of cistrons in the network module and the consensus sequence detected in all its members is given. Click here for file

Additional file 8 **Additional network modules enriched and with consensus sequence.** Network modules 20, 45, 636, and 691 enriched in a protein class and a GO term and containing a consensus sequence in all of its members (see Table 2** and additional file **7**)**. Click here for file
