# Supplementary material for: Genome-wide inference of regulatory networks in Streptomyces coelicolor
Source: BMC Genomics. 2010 Oct 18;11:578. doi: 10.1186/1471-2164-11-578 (PMC3224704; doi:10.1186/1471-2164-11-578)

Additional network modules enriched in a protein class and a GO term and with a consensus sequence in all its members. Node shapes and colors are as indicated in Figure 2.

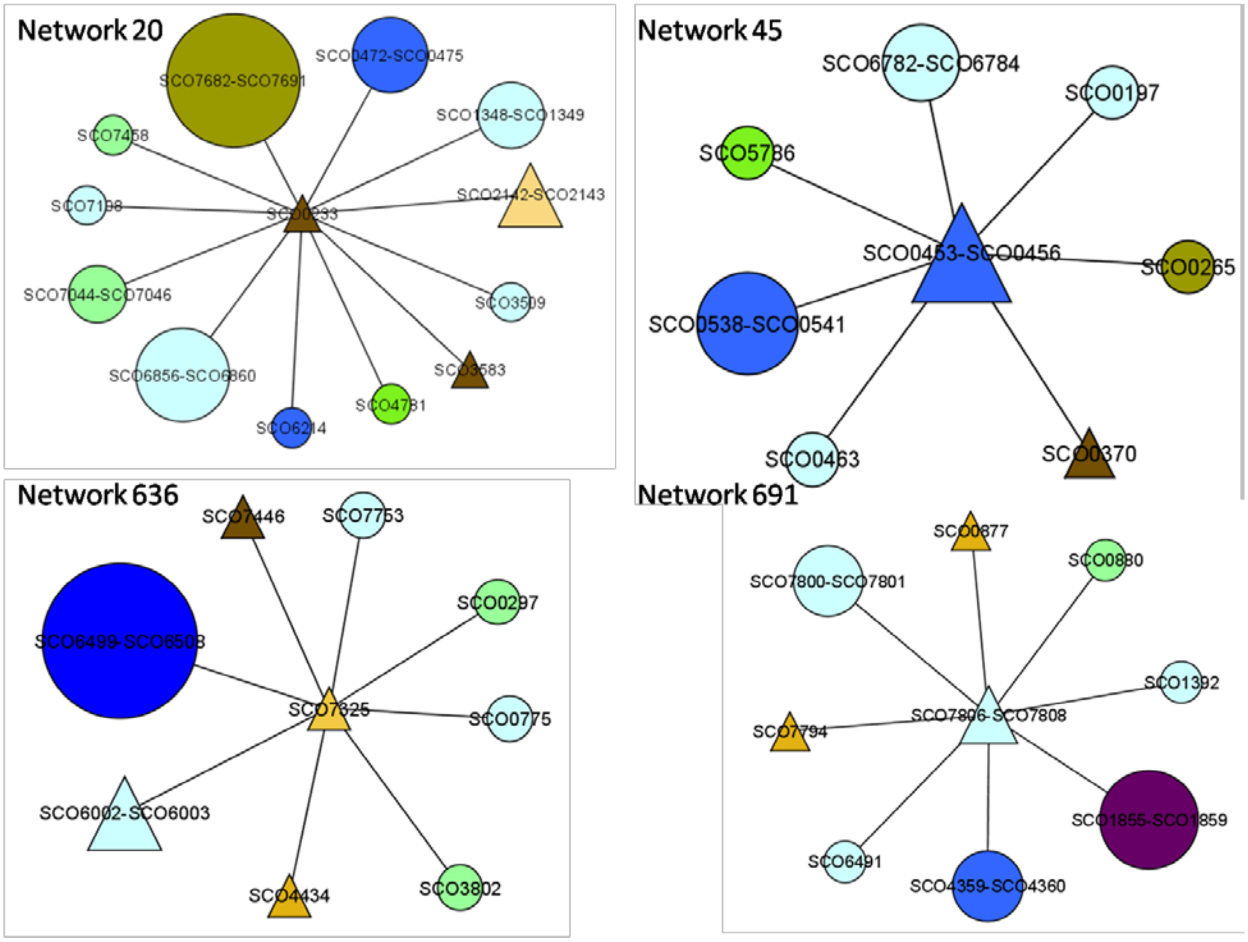

Supplement: Additional file 8 — Additional network modules enriched and with consensus sequence. Network modules 20, 45, 636, and 691 enriched in a protein class and a GO term and containing a consensus sequence in all of its members (see Table 2 and additional file 7). [file 1471-2164-11-578-S8.PDF]
